# Different construction strategies affected on the physiology of *Pichia pastoris* strains highly expressed lipase by transcriptional analysis of key genes

**DOI:** 10.1080/21655979.2019.1614422

**Published:** 2019-05-13

**Authors:** Jinjin Huang, Qing Wang, Wei Bu, Lingxiao Chen, Zhen Yang, Weifa Zheng, Ying Li, Jilun Li

**Affiliations:** a Key Laboratory for Biotechnology on Medicinal Plants of Jiangsu Province, School of Life Sciences, Jiangsu Normal University, Xuzhou, P. R. China; b State Key Laboratory of Agrobiotechnology and MOA Key Laboratory of Soil Microbiology, College of Biological Sciences, China Agricultural University, Beijing, P. R. China; c School of Life Sciences, Beijing University of Chinese Medicine, Beijing, P. R. China

**Keywords:** *Rhizomucor miehei* lipase, *Pichia pastoris*, propeptide, signal peptide, gene dosage, protein synthesis and secretion pathway

## Abstract

We demonstrated previously that expression of *Rhizomucor miehei* lipase (RML) in *Pichia pastoris* could be significantly increased by addition of gene propeptide, optimized signal peptide codons and manipulation of gene dosage. In this study, effects of various strategies on the protein synthesis and secretion pathways were analyzed. Using nine strains previously constructed, we evaluated cell culture properties, enzymatic activities, and analyzed transcriptional levels of nine genes involved in protein synthesis and secretion pathways by qPCR. We observed that (i) Addition of propeptide decreased lipase folding stress by down-regulated four UPR-related genes. (ii) Signal peptide codons optimization had no effect on host with no change in the nine detected genes. (iii) Folding stress and limited transport capacity produced when *rml* gene dosage exceed 2. Different limiting factors on lipase expression in strains with different construction strategies were identified. This study provides a theoretical basis for further improving RML by transforming host.

## Graphical Abstract

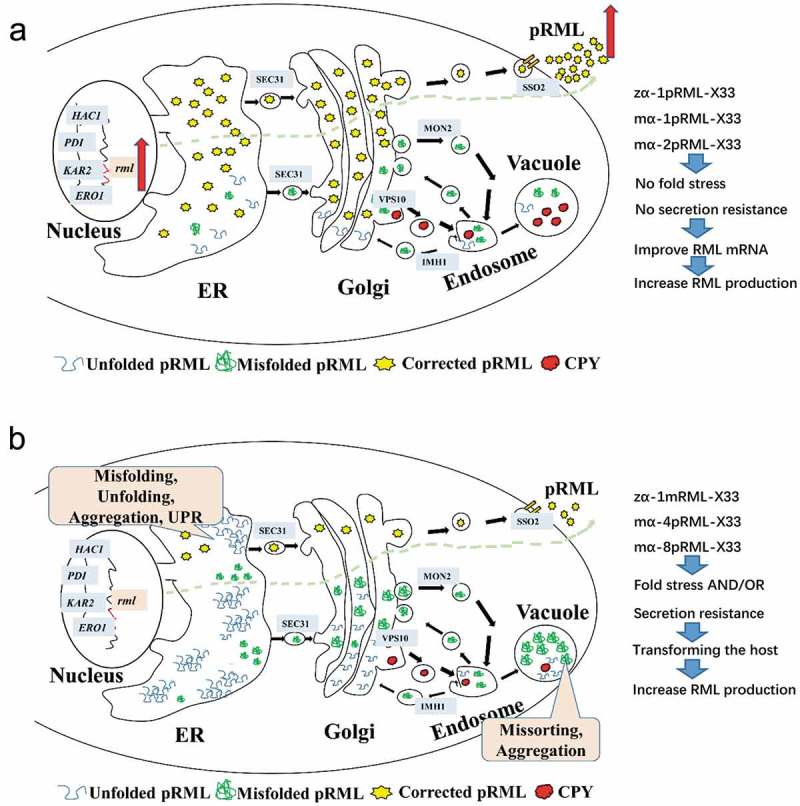

## Introduction

The methylotrophic yeast *Pichia pastoris* is one of the most widely used foreign protein expression systems for heterologous protein production [1]. Large amounts of proteins can be produced using this protein expression system [2,3]. Numerous studies explore ways to increase protein yield, such as optimizing gene codons, gene dosage, culture conditions, change promoters and host transformation, etc [4,5]. Among these strategies, modification of host to enhance the yield of foreign proteins is getting more and more attention. Modification of host may increase the yield of foreign proteins, however, sometimes not. For example, overexpression of protein disulfide isomerase (PDI) increased the production of mammalian peptidoglycan recognition proteins (PGLYRP-1) in high copy pglyrp-1 clones, but co-expression of binding protein (BiP) decreased PGLYRP-1 secretion [6]. In addition, study found that helper factors, such as Ero1, Sec53, and Cne1, did not improve *Rhizopus oryzae* lipase (ROL) activity in *P*. *pastoris* [7]. Strategies for optimizing protein yield are random because of uncertain the limiting factors, and rarely study on the relationship between physiological of host and protein yields used various strategies when expression of heterologous protein.

Major research progress on mechanisms of endogenous protein secretion has occurred during the past decade. In regard to the field of intracellular trafficking, the 2013 Nobel Prize in Physiology or Medicine was awarded to three of its most distinguished scientists (James Rothman, Randy Schekman, and Thomas Sudhof) for their work on vesicular transport mechanisms [8]. Protein synthesis and secretion mechanism is conserved in cell, such as unfolded protein response (UPR) [9] and quality control systems (QC systems) [10]. Unfolded or misfolded proteins accumulate in the endoplasmic reticulum (ER) through various reasons are toxic to cells, UPR is triggered by the accumulated misfolded proteins in ER, which also called ER stress or folding stress [11,12]. UPR response to maintain the steady state of ER [13,14] by enhancing the folding ability of unfolded or misfolded proteins in ER [15] and reducing new prepeptides enter to ER [16,17]. *HAC1, KAR2, PDI* and ERO1 genes can be used as the marker genes, and their mRNA levels are used for determining whether UPR is triggered [18].

In addition, there are strict QC systems in cell to ensure that only the right protein can be synthesized and secreted to perform their function, including ER-associated degradation (ERAD) [19], Golgi–endosomal-vacuolar/lysosome pathway [20–22], Golgi-plasma membrane-vacuolar/lysosome pathway [23] and so on. The protein is firstly transported to Golgi by vesicle transport (SEC31 was one of the key vesicle-forming proteins) [24], then the corrected protein is transported to plasma membrane by vesicles. Finally, the vesicles fused with plasma membrane through t-SNARE (*SSO2* coded) [25], and the target protein is secreted into the extracellular. But if the uncorrected protein appeared in the cell, the Golgi QC system will act to move the wrong protein into endosome and vacuole for degrading (*MON2* coded scaffold protein and located on the reverse side of the Golgi apparatus, involved in vesicle formation and transport uncorrected protein from the Golgi to the endosome) [26]. The amount of carboxypeptidase Y transported to the endosome will also increase (*VPS10* encoded vacuolar sorting proteins) [27]. Sometimes, uncorrected protein can be reversely transported from endosomes to Golgi for reprocessing into the correct protein and IMH1 involved in the process [28]. If transcript levels of those genes changed meaning protein can't be successfully secreted to the extracellular and existed transport stress.

Those discoveries provided important reference information for expression of foreign protein. UPR response had been found triggered when exogenous proteins overexpressed [29]. However, the steps in protein synthesis and secretory pathway in the host, in addition to protein folding in ER, also include immature protein transport from ER to Golgi, further processing in Golgi, and transport from Golgi to plasma membrane, etc. There are numerous factors at various steps in protein synthesis and secretion pathway that may potentially inhibit exogenous protein expression. To overcome such factors, and improve target protein yield, we need to better understand the changes that occur in the host during foreign protein expression.

*Rhizomucor miehei* lipase (RML) is a widely used biocatalyst in food, medicine, bioenergy, which is a single-chain α/β type protein. The mature enzyme (RML) is comprised by 269 amino-acid residues with the molecular weight (MW) 31.6 KDa [30], and found the lipase firstly formed a precursor (pRML) in the cell before the mature lipase produced. The precursor lipase includes a 70 amino-acid propeptide before the 269 amino-acid residues of the mature enzyme and its MW is 43 kDa [31]. In our 2014 study, we achieved ~21.4-fold increase in RML production in *P. pastoris* by adding the target gene propeptide, optimizing the signal peptide, and varying the number of target gene copies [32]. The enzyme (termed “Lipase GH2”) was effectively applied for conversion of microalgae biodiesel [33]. Extracellular lipase activity was highest in the recombinant strain containing two copies of *prml* gene (“2-copy strain”). The highest *prml* transcriptional level was observed for the 4-copy strain; however, in a comparison of transcriptional levels of four UPR-related genes (*HAC1, KAR2, PDI*, and *ERO1*) in ER between 2-copy and 4-copy strains, protein folding stress was found stronger in 4-copy strain than that in 2-copy strain [32].

In order to better understand the changes that occurred in the host when foreign protein expression, in the present study, we examined the variation on the steps (protein folding in ER, transport from ER to Golgi, further processing in Golgi, transport from Golgi to membrane) of protein synthesis and secretion pathway in recombinant strains constructed with different strategies previously. This study not only give us a better understanding of the changes in host for every strategy which gradually increased the production of RML, but also supply a theoretical basis for the next step of transforming strains to improve enzyme activity and provide reference for the efficient expression of other foreign genes (including other enzymes) in host such as *P*. *pastoris*.

## Materials and methods

### Strains used in this study

Nine strains (X-33, zα-X33, mα-X33, zα-1mRML-X33, zα-1pRML-X33, mα-1pRML-X33, mα-2pRML-X33, mα-4pRML-X33 and mα-8pRML-X33) used in this research were all constructed, fermented, and described in our 2014 study [32]. The strains and its RML production parameters are shown in Table S1.

### RNA extraction and cDNA synthesis

Each of the nine strains was flask-cultured in BMGY/BMMY medium, and target protein expression was induced every 24 h [32]. Samples for total RNA extraction were taken at 48 h and 96 h. Trizol reagent (Tiangen Biotech Co., Beijing, China) was used to isolate total cellular RNA [34]. M-MLV reverse transcriptase (Promega, Madison, WI, USA) was used to reverse transcription of RNA into cDNA [34].

### Real-time quantitative PCR

Transcriptional levels of nine genes (*HAC1, KAR2, PDI, ERO1, SEC31, SSO2, MON2, VPS10, IMH1*) in protein synthesis and secretion pathway were analyzed. The possible function of selected genes and primers with the efficiency ranged between 95% and 100% used for relative quantification by qPCR are listed in Table S2 and S3.

LightCycler 480 RT-PCR system was used in this study for real-time quantitative PCR. LightCycler 480 SYBR Green I Master Kit (Roche, Mannheim, Germany) was used as fluorescent dyes according to the manufacturer’s instructions. The glyceraldehyde-3-phosphate dehydrogenase gene (*gap*) of *P. pastoris* was used as reference gene. The comparative crossing point (CP) method and presented as 2^−∆∆Cp^ was used to analyze the relative expression of each gene. Three replicates were set up in this experiment and the data of the three replicates were averaged for next analysis.

The target gene copies were determined by absolute quantification as described in Huang et al. (2014)[32].

### Target lipase purification by Ni-NTA

Fermentation supernatant was obtained by centrifugation, and 10× buffer (500 mM NaH_2_PO_4_, pH 8.0, 1.5 M NaCl, 100 mM imidazole) (1/10 volume) was added to the medium. pH of the mixture was maintained at 8.0. The supernatant was mixed with 1 mL Ni-NTA agarose (part # 30,210, Qiagen, Germany) to conjugate protein. Protein was eluted with 3 mL buffer (50 mM Tris-HCl, pH 8.0, 300 mM NaCl) containing successive concentrations of 50, 80, 100, 150, 200, and 250 mM imidazole. Protein concentration was quantified by Bradford assay [34], with bovine serum albumin (BSA) as standard.

### Enzyme characterization

Lipase hydrolysis activity was carried out by NaOH titration method [32]. 45°C and 0.1M Tris-HCl pH 8.0 were used for RML, and 35°C and 0.1 M sodium dihydrogen phosphate/citric acid pH 6.0 for pRML. Protein concentration was quantified by Bradford assay [35]. Specific activity-N (U/mg）was calculated from the ratio of the enzyme activity (U/mL) to the protein concentration (mg/mL) of the purified protein. Extracellular lipase concentration-N (mg/mL) was calculated from the ratio of extracellular enzyme activity (U/mL) to specific activity-N (U/mg). Extracellular lipase activity secretion efficiency (U/OD_600_) was calculated from the ratio of extracellular enzyme activity (U/mL) to cell growth (OD_600_). Lipase protein secretion efficiency (mg/OD_600_) was calculated from the ratio of extracellular lipase concentration-N (mg/mL) to cell growth (OD_600_). The extracellular enzyme activity (U/mL) and cell growth (OD_600_) are shown in Table S1.

### Intracellular target protein detection by Western-blot

The precipitate for each strain was centrifuged, washed three times with sterile distilled water, and diluted to a defined cell concentration (OD_600_ = 1), 40 μL of the mixture was added with 10 μL of 5× loading buffer (250 mM Tris-HCl, pH 6.8, 10% (w/v) SDS, 0.5％(w/v) BPB, 50％(v/v) glycerin, 5％(w/v) β-mercaptoethanol), boiled for 5 min, and 20 μL samples were subjected to Western blotting. 5% stacking gel and 12% resolving gel was used in sodium dodecyl sulfate polyacrylamide gel electrophoresis (SDS-PAGE). Monoclonal mouse-anti-His-Tag antibody (Tiangen, Beijing, China) and goat anti-mouse IgG antibody conjugated to alkaline phosphatase (Sigma-Aldrich, St. Louis, MO, USA) were used as the primary antibody and the secondary antibody. BCIP/NBT Chromogenic reagent kit (Tiangen) was used for color reaction according to the manufacturer’s instructions.

### Hierarchical clustering analysis

To evaluate the expression trend  of target gene involved in protein synthesis and secretion pathway of different strains, hierarchical clustering (HCL) was performed and a distance tree was generated using the multi-experiment viewer (MeV) program according to Wang et al.(2013) [34]. A log_2_ transformation of an expression fold change between control strain and sample strain was used for each expression element.

## Results

### Propeptide can eliminate ER stress by downregulated four UPR-related genes and no triggered transport pressure

**Figure 1.**
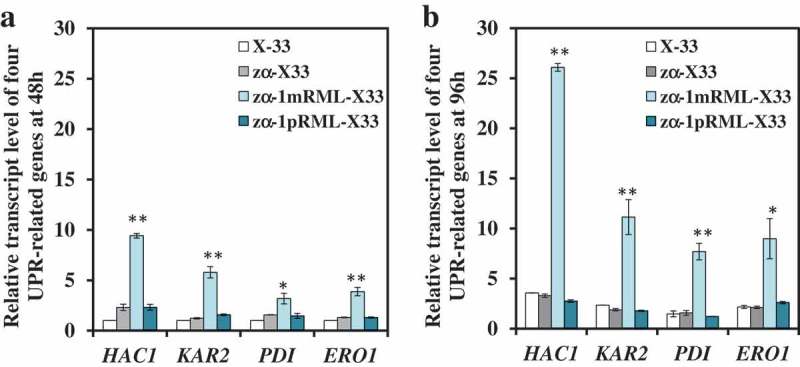
Comparison of the transcript levels of four UPR-related genes in RML-expressed strain before and after adding propeptide. Levels of UPR-related genes (*HAC1, KAR2, PDI* and *ERO1*) in X-33 at 48 h were used as reference for statistical analysis of differences by Student’s *t*-test. * *P* < 0.05. ** *P* < 0.01. Three replicates were set up in this experiment and the data was averaged from three replicates.(a): Relative transcript levels of UPR-related genes at 48 h. Compared with X-33, all the transcript level of four UPR-related genes were up-regulated in zα-1mRML-X33, and no significant changes compared with that in X-33 after adding propeptide in zα-1pRML-X33 at 48 h.(b): Levels of UPR-related genes at 96 h.Levels of *HAC1, KAR2, PDI* and *ERO1* were all up-regulated in zα-1mRML-X33 and no significant changes in zα-1pRML-X33 at 96 h.

A propeptide with 70 amino-acid was added before RML when used *P*. *pastoris* X-33 as heterologous protein expression host [32]. And it found that the extracellular lipase activity and the content of extracellular protein of recombinant strain with propeptide (zα-1pRML-X33, 430 U/mL, 0.15 mg/mL) were all improved ~7-fold than that strain without propeptide (zα-1mRML-X33, 56 U/mL, 0.019 mg/mL) in flask fermentation, and the transcription level of *prml* improved about 1–1.5 times (Table S1) [32]. Now, we want to know how did the propeptide promote enzyme activity, increase protein content in extracellular and restore the cell growth (Table S1). In order to answer the questions above, relative transcription levels of nine genes (*HAC1, KAR2, PDI, ERO1, SEC31, SSO2, MON2, VPS10, IMH1*) participated in different process of protein synthesis and secretion pathway were analyzed in this study before and after adding propeptide when fermented at 48 h and 96 h. The result of the relative transcript levels of the nine detected genes were shown in Figure 1 and Figure S1. It was found that only the transcript levels of four UPR-related genes (*HAC1, KAR2, PDI*, and *ERO1*) were all up-regulated in the strain without propeptide (zα-1mRML-X33), but down-regulated to normal level in the strain adding propeptide whether it was fermented at 48 h or 96 h (Figure 1). However, other genes didn't have significant change in transcriptional level (Figure S1). This proved that propeptide can reduce the folding pressure of pRML in ER. Propeptide can help pRML effectively fold and the correct lipase protein could be successfully secreted into the extracellular without triggered transport pressure. This is another reason for the extracellular lipase activity improved besides the increase in transcription level of *prml*. The cellar physiology of zα-1pRML-X33 returns to normal level after adding propeptide, which is the reason for the cell growth of zα-1pRML-X33 consistent with the control strain zα-X33.

### Signal peptide codons optimization didn’t trigger ER stress and protein transport stress

**Figure 2.**
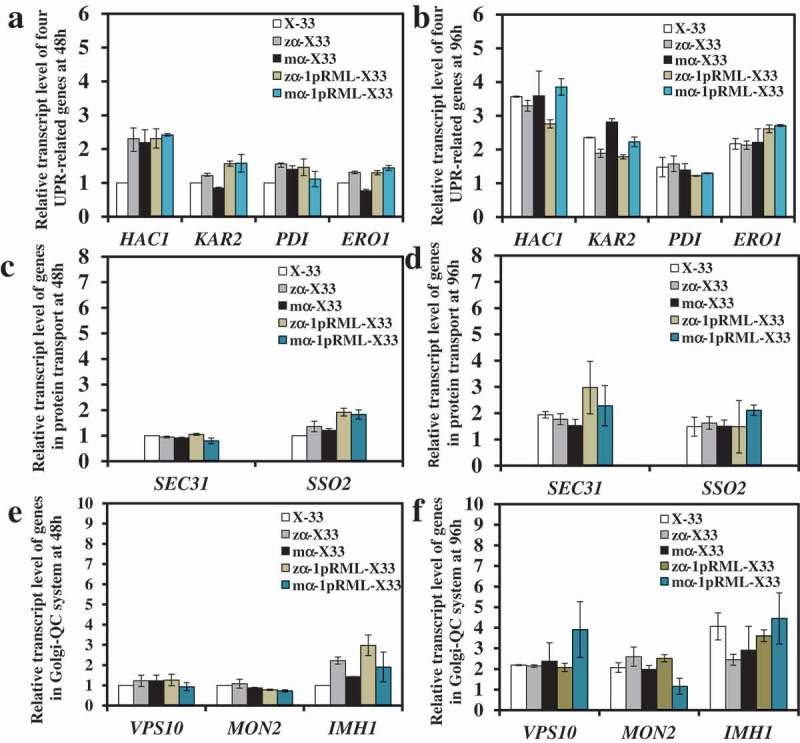
Comparison of the transcript levels of nine genes participated in UPR, protein transport and Golgi-QC system before and after optimizing signal peptide codons. Levels of genes in X-33 were used as reference for statistical analysis of differences by Student’s *t*-test. * *P* < 0.05. ** *P* < 0.01. Three replicates were set up in this experiment and the data were averaged from three replicates.(a): Genes participated in UPR fermented at 48h; Levels of *HAC1, KAR2, PDI* and *ERO1* were all no significant changes in zα-1pRML-X33 and mα-1pRML-X33 at 48 h.(b): Genes participated in UPR fermented at 96 h. The same trend with 48 h at 96 h.(c): Genes participated in protein transport fermented at 48h; Levels of *SEC31* and *SSO2* were all no significant changes in zα-1pRML-X33 and mα-1pRML-X33 at 48 h.(d): Genes participated in protein transport fermented at 96 h. The same trend with 48 h at 96 h.(e): Genes participated in Golgi-QC system fermented at 48 h. Levels of *VPS10, MON2* and *IMH1* were all no significant changes in zα-1pRML-X33 and mα-1pRML-X33 at 96 h.(f): Genes participated in Golgi-QC system fermented at 96 h. The same trend with 48 h at 96 h.

The extracellular lipase activity and the content of extracellular protein of recombinant strain with optimizing signal peptide codons (mα-1pRML-X33, 600 U/mL, 0.33 mg/mL) were all improved ~1.4-fold than that strain without optimizing signal peptide codons (zα-1pRML-X33, 430 U/mL, 0.15 mg/mL) in flask fermentation, and the improvement in extracellular lipase activity was consistent with the enhancement (~1.4 fold) in transcriptional level of *prml* (Table S1) [32]. So we theorized that signal peptide codons optimization didn't cause protein folding stress in ER and protein secretion resistance. In order to prove our inference, transcriptional levels of nine key genes were detected among the strains X-33, zα-X33, zα-1pRML-X33, mα-X33, and mα-1pRML-X33 cultured at 48 h and 96 h by RT-qPCR. The result was shown in Figure 2. The result found that all the nine genes were no significant change in transcriptional level compared with control strain X-33. No change in the transcription levels of four UPR-related genes proved no ER stress. The increased mRNA of pRML in mα-1pRML-X33 can be correctly folded. At the same time, no change in the transcription levels of *SEC31, MON2, VPS10, IMH1*, and *SSO2* genes indicated no protein secretion stress. Those all illustrated that no ER stress and protein transport stress in strain after optimized signal prptide, and the increased *prml* mRNA in ma-1pRML-X33 can be properly folded and successfully secreted into extracellular . The enhancement of extracellular enzyme activity in mα-1pRML-X33 was consistent with the improvement of transcription level of lipase gene, and the cell growth was also didn't been affected.

### Different with 4-copy strain gradually triggered ER stress, 8-copy strain triggered ER stress only in 48h, but removed it in 96h

The extracellular enzyme activity of the strains with 1-copy, 2-copy, 4-copy and 8-copy were 600, 1200, 713 and 503 U/mL, respectively (Table S1) [32], and more protein folding stress was found in 4-copy strain than 2-copy strain by comparison of transcriptional level change of four UPR-related genes between 2-copy and 4-copy strain at 96 h [32]. However, there are other questions needed to be discussed: (1) whether the folding stress and limited transport capacity also produced in 2-copy strain? (2) How many copy number of the *prml* increase to, it began to produce pressure in the host, 1-copy, 2-copy or 4-copy strain? (3) Whether 8-copy strain produced the pressure in host? Is the pressure same as 4-copy?

**Figure 3.**
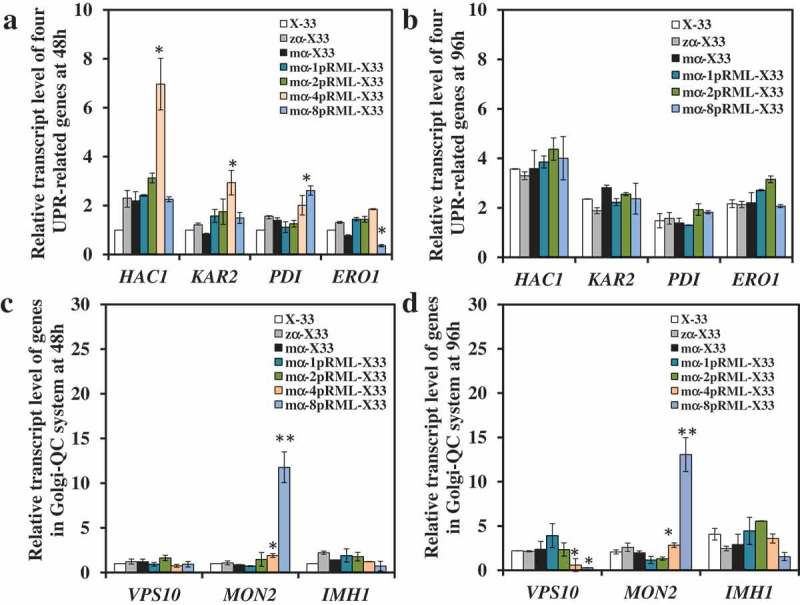
Comparison of the transcript level of genes participated in UPR and Golgi-QC system in the strains with different lipase gene dosage. Levels of genes in X-33 were used as reference for statistical analysis of differences by Student’s *t*-test. * *P* < 0.05. ** *P* < 0.01. Three replicates were set up in this experiment and the data were averaged from three replicates. (a): Genes participated in UPR fermented at 48h; Levels of *HAC1* and *KAR2* was significantly up-regulated in 4-copy strain, while didn't change in 8-copy stain at 48h. *PDI* was up-regulated and *ERO1* was down-regulated in 8-copy strain, but all the two genes with no obvious changes in 4-copy strain at 48h. (b): Genes participated in UPR fermented at 96h; Levels of genes were all didn’t change in 1-, 2- and 8-copy strain. (c): Genes participated in Golgi-QC system fermented at 48 h. Levels of *MON2* was up-regulated in 4- and 8-copy strain, while others didn't change at 48 h. (d): Genes participated in Golgi-QC system fermented at 96 h. Levels of *VPS10* was down-regulated and *MON2* was up-regulated in 4-copy and 8-copy strain at 96 h.

In order to answer the questions above, gene dosage affected on pRML synthesis and secretion pathway in *P*. *pastoris* was investigated by analyzing the transcript level change trend of nine genes. Transcriptional levels of four UPR-related genes in 1-copy, 2-copy,4-copy and 8-copy strains used X-33 as control strain were compared at 48 h (Figure 3(a)). But only 1-copy, 2-copy and 8-copy strains were compared at 96 h (Figure 3(b)). Transcript levels of four UPR-related genes at 96 h in 4-copy strain didn't shown in Figure 3(b), because it had been compared in Huang et al.(2014) and found protein folding stress was stronger in 4-copy strain than that in 2-copy strain [32]. All the transcriptional levels of four UPR-related genes were no obviously change when gene dosage increased from 1 to 2 at 48 h and 96 h, but there was clear up-regulation in 4-copy of *HAC1* and *KAR2* at culture stage (48 h) when gene dosage increased to 4 at 48 h (Figure 3(a)). Simultaneously, all four UPR-related genes in 4-copy was found up-regulation than that in 2-copy strain at 96 h [32]. These results indicated that ER stress in 4-copy was gradually increased as protein production improved. When gene dosage increased to 8, there was no change in transcriptional levels of *HAC1* or *KAR2* at culture stage (48 and 96 h), whereas *PDI* was up-regulated ~2-fold at 48 h. *ERO1* was down-regulated ~2-fold at 48 h (Figure 3(a)) and both returned to control level at 96 h (Figure 3(b)). These findings indicated that misfolded or unfolded proteins accumulated in ER when the gene dosage exceeded 2. The protein fold pressure was different in 4- and 8-copy strains. In 4-copy strain, UPR was gradually triggered from the logarithmic phase to the stationary phase, but an increase of protein fold pressure in early-stage fermentation process in 8-copy, and removal of protein fold pressure in the late stage.

Transcriptional levels of *SEC31, SSO2, IMHI, VPS10*, and *MON2* on the four strains at 48 and 96 h used X-33 as control strain were also been studied. As the gene dosage increased from 1 to 8, no changes were found in transcript levels of *SEC31, SSO2*, and *IMHI* at 48 h and 96 h (Figure S2 and Figure 3(c,d)). For *VPS10* and *MON2*, no changes in 1-copy and 2-copy strains at 48 or 96 h (Figure 3(c,d)). But *VPS10* transcriptional level was down-regulated only at 96 h in 4-copy and 8-copy strains (Figure 3(c,d)). In contrast, *MON2* transcriptional level was significantly up-regulated at 48 and 96 h in 4-copy and 8-copy strains (Figure 3(c,d)). Those results suggested that protein secretion pressure was similar in 4-copy and 8-copy strain. A large number of misfolded or unfolded proteins were transported to vacuoles and accumulated in vacuoles, but the amount of carboxypeptidase Y (CPY) which can degrade error protein in vacuoles was reduced. This suggested that many proteins were presumably identified as error proteins and sorted to vacuoles for degradation, resulting in accumulation of proteins in 4-copy an 8-copy.

### Intracellular target protein in strains with different gene dosage

**Figure 4.**
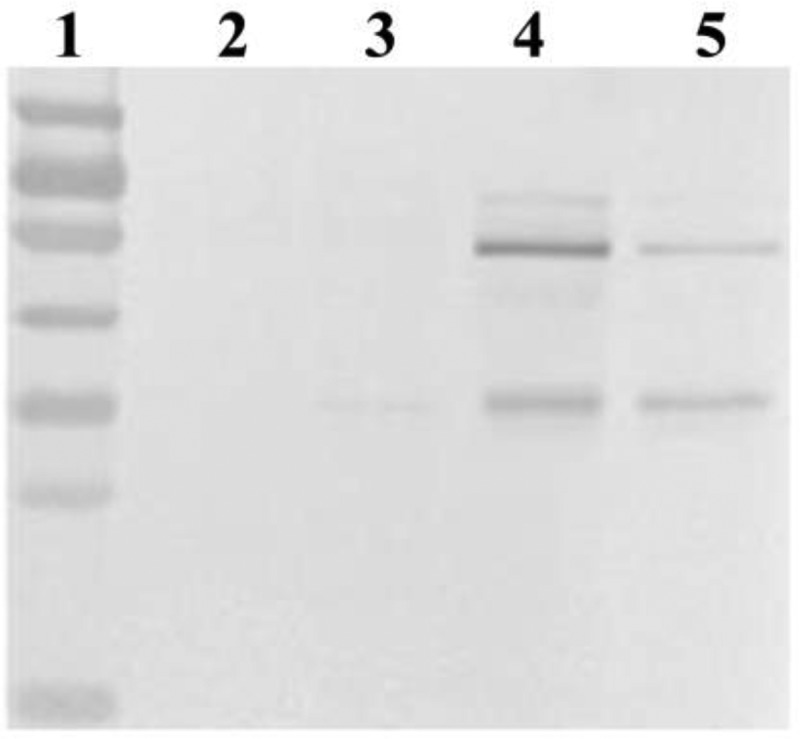
Detection by Western blotting of intracellular lipase in four recombinant strains. Lane 1: protein markers (top to bottom: 100, 70, 55, 40, 35, 25, 15 kDa). Lanes 2–5: intracellular protein (lipase) detected in the four strains.Little or no target protein (32-kDa protein without propeptide, or 55- to 70-kDa protein containing propeptide) was detected in intracellular compartment of 1- or 2-copy, whereas some target protein was retained in 4- and 8-copy.

Intracellular target protein of different strains with 1, 2, 4 and 8 copies was detected in this study and the result was shown in Figure 4. It was found that no intracellular lipase was detected in 1-copy and 2-copy strains (Figure 4, Lane 2 and 3), but intracellular protein accumulation was produced when the copy number of *prml* exceed 2 (Figure 4, Lane 4 and 5).

### The characteristic of pRML secreted by strains with different gene dosage

Secreted target lipase was purified from fermentation supernatant using Ni-NTA agarose and the parameters recalculated with purified lipase were shown in Table 1. In order to distinguish from the parameters in previously study which were calculated by extracellular total protein in fermentation broth [32], specific activity and protein concentration calculated with purified protein by Ni-NTA in this study were renamed as specific activity-N and protein concentration-N. Different from the date in the 2014 study, specific activity-N of the four strains (1-copy, 2-copy, 4-copy and 8-copy) were 2718, 2298, 1110 and 928 U/mg, respectively. The specific activity-N of 1- and 2-copy strain was more higher than the specific activity in 2014 study (1543 U/mg and 1684 U/mg), while the specific activity-N of 4- and 8-copy strain was similar than the specific activity in previously study (~1018 U/mg and 707 U/mg). However, in this study, extracellular lipase concentration-N (mg/mL) was gradually increased as 0.15 mg/mL, 0.35 mg/mL and 0.51 mg/mL when the copy number was 1, 2, and 4, but reduced to 0.24 mg/mL when the copy number increased to 8. The trend was different with extracellular total protein content detected in 2014 study, which proved extracellular protein was increased when the copy number from 1(0.3 mg/mL) to 2 (0.77 mg/mL), then basically unchanged even increase to 4 (0.69 mg/mL) and 8(0.73 mg/L) [32]. Extracellular lipase activity secretion efficiency (U/OD_600_) was defined as the ratio of extracellular enzyme activity (U) to optical density (OD_600_). Values of this parameter for the four strains were, respectively, 9.33, 19.96, 18.86, and 8.33, i.e. the value was highest for 2-copy strain. Lipase protein secretion efficiency (mg/OD_600_) was similarly defined as the ratio of extracellular target protein concentration (mg) to optical density (OD_600_). Values of this parameter for the four strains were, respectively, 0.003, 0.009, 0.017, and 0.009 (Table 1), i.e. the value was highest for 4-copy strain.

**Table 1.** Parameters of lipase produced by recombinant strains containing different copy number of the *prml* gene.

Copy number	1	2	4	8
Specific activity-N (U/mg)	2718 ± 67	2298 ± 412	1110 ± 143	928 ± 54
Extracellular lipase concentration-N (mg/mL)	0.15 ± 0.01	0.35 ± 0.04	0.51 ± 0.00	0.24 ± 0.01
Extracellular lipase activity secretion efficiency (U/OD_600_)	9.33 ± 0.40	19.96 ± 2.17	18.86 ± 0.00	8.33 ± 0.26
Lipase protein secretion efficiency (mg/OD_600_)	0.003 ± 0.0001	0.009 ± 0.0009	0.017 ± 0.0000	0.009 ± 0.0003

## Discussion

Now many studies were used to improve production of heterologous protein by optimizing gene dosage or modifying the host, but not all can increase the expression of exogenous proteins. In most cases, increasing the gene dosage will increase protein production, however, increasing the gene dosage *pglyrp-1* decreased its production [6]. UPR modification is the most concerned way to improve the expression of exogenous proteins in *P*. *pastoris*. However, not every UPR-related gene modification can increase the expression of foreign proteins. For example, overexpression of *KAR2* in *P*. *pastoris* had no effect on the expression level of 2F5mAb in humans [31]. Some studies have found that overexpression of protein folding-related genes (*PDI, ERO1, KAR2, IMH1, SEC31*) increases expression levels of foreign proteins [14,28,36]. On the other hand, Samuel et al. reported that expression of *KAR2* gene in *P. pastoris* caused a 0.7-fold reduction of *Candida antarctica* lipase B extracellular expression level [37]. It is also indicated that the restriction factors of different strains are different, and the strategies adopted should also be different. Limiting factors differ for recombinant strains, produced using different construction strategies, even differing copy numbers of a particular gene. However, most of the current studies focus on UPR, even if not all strains are suitable for UPR modification. It is therefore important to distinguish strategies for modification of different hosts.

In previously study, we significantly enhanced the RML production in *P. pastoris* by different strategies and obtained strains with different levels of enzyme production [32]. In the present study, we performed a comprehensive analysis of the transcriptional levels of nine genes involved in synthesis and secretion pathway to understand the changes that occurred in the pathway when used different strategies.

**Figure 5.**
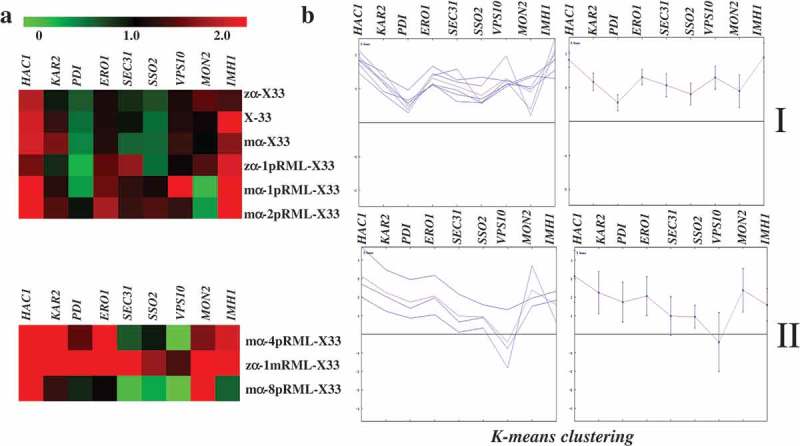
Transcription levels and HCL of nine target genes related to protein synthesis and secretion pathway in different strains at 96 h. (a): HCL of fold change patterns of nine target genes using the multi experiment viewer program, v 4.8.1. The expression matrix shows a false-color view on a red-green scale, with green representing low expression and red representing high expression.(b): *k*-means clustering of each gene and the overall trend from the HCL in (a).Those strains can be divided into two categories: Categories I with no fold pressure and transport resistance produced in host including X-33, zα-X33, mα-X33, zα-1pRML-X33, mα-1pRML-X33 and mα-2pRML-X33. Categories II with protein fold pressure and transport resistance produced in host including zα-1mRML-X33, mα-4pRML-X33 and mα-8pRML-X33.

When the protein is expressed, the pressure generated in different periods is different, but the later pressure is more obvious. Therefore, we clustered the transcription levels of each gene in the fermentation for 96 h. Expression trend of target gene involved in protein synthesis and secretion pathway of different strains at 96 h analyzed by HCL was shown in Figure 5, and found those strains can be divided into two categories: Categories I was X-33, zα-X33, mα-X33, zα-1pRML-X33, mα-1pRML-X33 and mα-2pRML-X33, the common point of those strains was no fold pressure and transport resistance were found. Categories II contained the strains of zα-1mRML-X33, mα-4pRML-X33 and mα-8pRML-X33, these strains were all detected protein fold pressure or transport resistance in host.

Combining the above categories with the enzyme production and the transcript level of target lipase gene, we found the lipase activity of strains in categories I (zα-1pRML-X33, mα-1pRML-X33 and mα-2pRML-X33) was progressively increased with the transcript level of target lipase gene step one by one [32]. In this study, no fold pressure and secretion resistance produced in those strains (Figures 2 and 3), and no intracellular target protein was detected in mα-1pRML-X33 and mα-2pRML-X33 (Figure 4), specific activity-N of pRML secreted by mα-1pRML-X33 and mα-2pRML-X33 was no obvious effected (Table 1). So the improved transcript level of *prml* can be smoothly folded and secreted into the extracellular in those strain. And with the extracellular lipase concentration-N, extracellular lipase activity secretion efficiency and lipase protein secretion efficiency was improved. Those proved that in the strains without fold pressure and secretion resistance, the transcription of the target lipase gene was the limited process in lipase production.

While other strains in Categories II (zα-1mRML-X33, mα-4pRML-X33 and mα-8pRML-X33) were all detected protein synthesis or secretion resistance (Figures 1 and 3), intracellular target protein was detected in mα-4pRML-X33 and mα-8pRML-X33 (Figure 4). The specific activity-N of pRML secreted by mα-4pRML-X33 and mα-8pRML-X33 was obviously reduced (Table 1). Fold pressure and secretion resistance were the main limited process instead of the transcription of the target lipase. Even if the transcript level of the target lipase is increased, it cann't be successfully folded and secreted. The change of extracellular lipase concentration-N, extracellular lipase activity secretion efficiency and lipase protein secretion efficiency in those strains were not consistent with the change of the transcription of the target lipase. Those proved that fold pressure and secretion resistance were the main reasons which decreased the lipase production in those strains. The idea of continuing to raise *prml* transcription level in order to express corrected lipase in this case did not make sense, because of the increased fold pressure and limited transport capacity.

In the present study, we found 4-copy strain had the highest *prml* mRNA, extracellular target protein and lipase protein secretion efficiency (mg/OD_600_), although reduction in extracellular enzyme activity (Table S1) and specific activity-N (Table 1). However, unfortunately, it triggered not only ER stress but also transport pressure. The idea of continuing to raise *prml* mRNA in order to express corrected lipase in this case did not make sense, because of the produced fold stress and limited transport capacity. Thus, fold stress and transport capacity were the limiting factors in 4-copy, rather than *prml* transcriptional level. The RML production may be further improved by modifying the host to eliminate or alleviate the above two limiting factors by modifying the host.

Improvement of expression level for any heterologous protein requires release or alleviation of stress on synthesis and secretion pathway in the host. An important step in this regard is construction of a library of host cells with modification (disruption or overexpression) of target genes, to allow adaptation to expression of a wide variety of foreign genes. This study would not only help to get a better understanding of three strategies effect on recombinant *P. pastoris,* but also give the ideas for further improving pRML production by modified4-copy strain to help the increased *prml* mRNA folded and secreted to extracellular. Enhanced expression of a foreign gene needs suitably match with the protein expression and transport system of host. Next, we will continue to carry out this work to further reduce production cost of pRML.

## Conclusion

Culture properties, enzymatic activities, and transcriptional levels of nine genes involved in protein synthesis and secretion pathway of *P. pastoris* were analyzed in recombinant strains expressed RML. The reasons why different construction strategies affected on the lipase production of *P*. *pastoris* strains were determined. The major limiting factors (transcription of the target lipase gene, fold pressure, or/and limited protein transport) were observed for affecting the production of lipase in *P. pastoris*. Lipase expression can be improved by overcoming these limiting factors in the host. In the strains without fold pressure and secretion resistance, the production of enzyme can be increased by improving the transcription of the target lipase gene, while the production of enzyme dropped when the transcription of the target lipase gene exceeded the capacity of protein synthesis and secretion in host, which caused fold pressure or/and protein transport pressure. Based on preliminary analysis of host cell protein synthesis and secretion pathways, 4-copy strain had highest mRNA level of *prml* and extracellular lipase concentration-N but low correct protein content. Relieved fold pressure and protein transport pressure in 4-copy strain to help the highly expressed mRNA effectively form into correct protein and secrete it out of the cell will be used for next strategy to improve the production of RML.
