# Supplementary material for: Different construction strategies affected on the physiology of Pichia pastoris strains highly expressed lipase by transcriptional analysis of key genes
Source: Bioengineered. 2019 May 13;10(1):150–61. doi: 10.1080/21655979.2019.1614422 (PMC6527059; doi:10.1080/21655979.2019.1614422)
Supplement: Supplemental Material [file kbie-10-01-1614422-s001.docx]

| Strains and source | Description | Enzyme activity  (U/mL) | OD_600_ | Extracellular protein content (mg/mL) | Relative transcription level of *rml or prml* at 96h |
| --- | --- | --- | --- | --- | --- |
| X-33 | *Pichia pastoris* X-33 (WT Mut^+^) | 0 |  |  |  |
| zα-X33 | X-33 containing only pPICZα A | 0 | 46±0.98 |  |  |
| mα-X33 | X-33 containing only pPICMα A which derived from pPICZα A with optimizing codons of α-factor | 0 | 50±0.14 |  |  |
| zα-1mRML-X33 | One copy RML gene without propeptide used pPICZα A expressed in X-33 | 56 | 39±1.77 | 0.019 | 1 |
| zα-1pRML-X33 | One copy RML gene with propeptide used pPICZα A expressed in X-33 | 430 | 49±1.41 | 0.15 | 2.7±0.25 |
| mα-1pRML-X33 | One copy RML gene with propeptide used pPICMα A expressed in X-33 | 600 | 44±2.97 | 0.33 | 4.1±0.20 |
| mα-2pRML-X33 | Two copies RML gene with propeptide used pPICMα A expressed in X-33 | 1200 | 41±4.67 | 0.77 | 9.4±1.18 |
| mα-4pRML-X33 | Four copies RML gene with propeptide used pPICMα A expressed in X-33 | 713 | 30±2.33 | 0.69 | 14.8±0.79 |
| mα-8pRML-X33 | Eight copies RML gene with propeptide used pPICMα A expressed in X-33 | 503 | 27±3.96 | 0.73 | 8.0±0.39 |

Table S1 The strains and its RML production parameter^31^

Note: The strains and date were determined in Huang *et al* (2014)^32^.

|  | Gene | GenBank accession number | Description |
| --- | --- | --- | --- |
| UPR | *HAC1* | FR839628 | bZIP transcription factor that regulates UPR^32^ |
|  | *KAR2* | XM_002490982 | BiP, a major Hsp70 chaperone in the ER (Sc), an ATPase regulates UPR; involved in ER quality control and ER-associated degradation^31^ |
|  | *PDI* | ACF17572 | Protein disulfide isomerase (PDI)^32^ |
|  | *ERO1* | XP_002489645 | PDI oxidase; glycoprotein required for oxidative protein folding in the ER, protein thiol-disulfide exchange^32^ |
| Vesicle transport from ER to Golgi | *SEC31* | CCA37204 | COPII coat of secretory pathway vesicle component (p150), involved in protein transport from ER to Golgi; structural molecule^24^ |
| Golgi quality control system | *MON2* | FR839629 | Golgi-to-endosome traffic, endocytosis, vacuole integrity^26^ |
|  | *VPS10* | FR839629 | Vacuolar protein sorting in Golgi membrane, late-Golgi^27^ |
|  | *IMH1* | CCA37012 | Protein involved in vesicular transport; mediates transport between an endosomal compartment and Golgi; contains a Golgi localization (GRIP) domain ^28^ |
| Vesicle and membrane fusion | *SSO2* | FR839628 | Secretory vesicle fusion with plasma membrane^25^ |

Table S2 The function of key genes in protein synthesis and secretion pathway

Table S3 The primers used for RT-qPCR

| Gene | Primer | Sequence 5′-3′ | Amplicon size (bp) |
| --- | --- | --- | --- |
| *gap* | *gap*-f | TACGTCATTGAGTCCACCGGT | 184 |
|  | *gap*-r | TGGTAGTACAAGAAGCATTGGAG |  |
| *HAC1* | HAC1-f | CAAGAATCAGCCAAAGCC | 199 |
|  | HAC1-r | TGCGAGTGGATGTAGATGC |  |
| *KAR2* | KAR2-f | CCTACTTCAACGACGCTCAA | 199 |
|  | KAR2-r | CCACCCTCAATAGAAAGCAGA |  |
| *PDI* | PDI-f | GAGCAACAAGAAGTTTGGAGTTCC | 260 |
|  | PDI-r | CCTCATAAGCAGGAGCCATTC |  |
| *ERO1* | ERO1-f | CGTTAGCAAACCCTCAAATCC | 201 |
|  | ERO1-r | GCAGAATCCCTCATCACCATT |  |
| *SEC31* | SEC31-f | CGTGTTCCTCAATCAGCCA | 198 |
|  | SEC31-r | TAGTTGCCTGGGTGAAGCA |  |
| *MON2* | MON2-f | GGGACTCTTTGGGTATTTCG | 249 |
|  | MON2-r | ATCGGGCTCACCATCTTA |  |
| *VPS10* | VPS10-f | TGAAGAATGGACGGGAAG | 221 |
|  | VPS10-r | GCTCACATCGGTTAGACTCTG |  |
| *IMH1* | IMH1-f | CGGATTCCAGTTTACTAAGCGA | 219 |
|  | IMH1-r | AGGGTGTAGCAGATGAGCGA |  |
| *SSO2* | SSO2-f | GTTCTCAACGGCTTTGCT | 194 |
|  | **SSO2-r** | **TGTCTACCAAGTTCTCGGTCT** |  |

Note: The primers were all used according to Huang *et al* (2014)^32^.
