# Supplementary figures and images for: Different construction strategies affected on the physiology of Pichia pastoris strains highly expressed lipase by transcriptional analysis of key genes
Source: Bioengineered. 2019 May 13;10(1):150–61. doi: 10.1080/21655979.2019.1614422 (PMC6527059; doi:10.1080/21655979.2019.1614422)

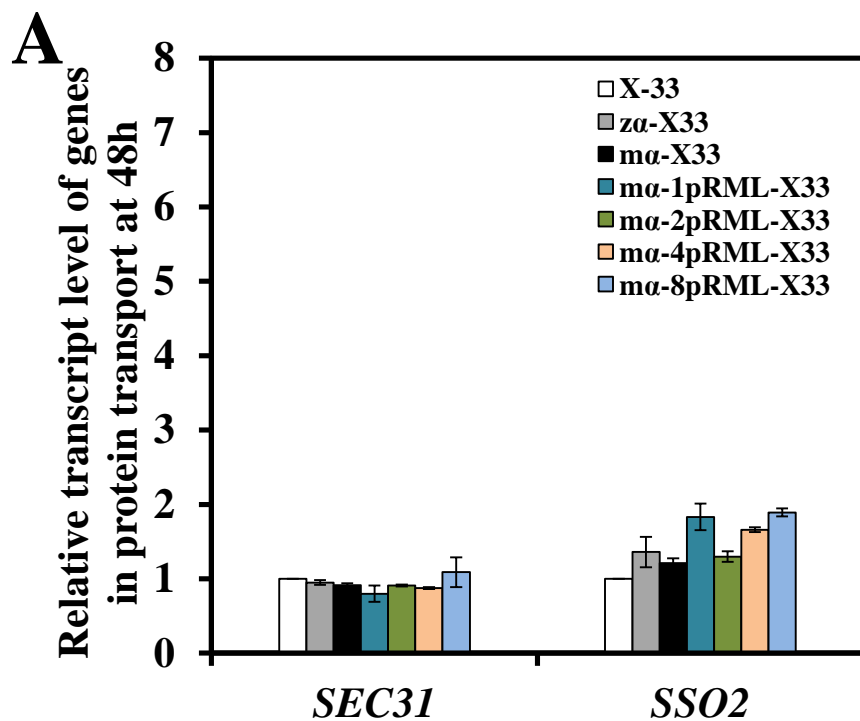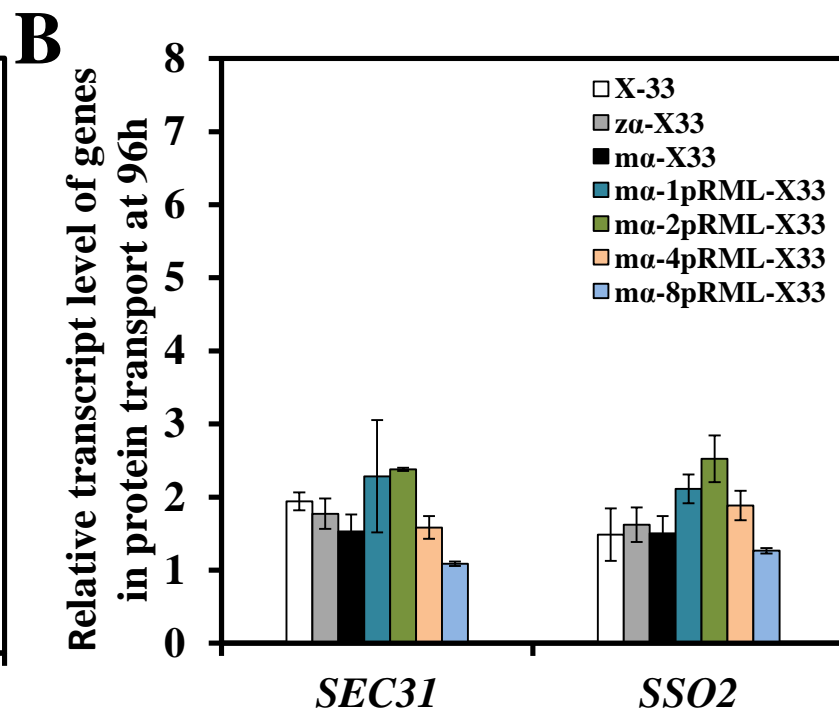

Supplement: Supplemental Material [file kbie-10-01-1614422-s002.pdf]

**A**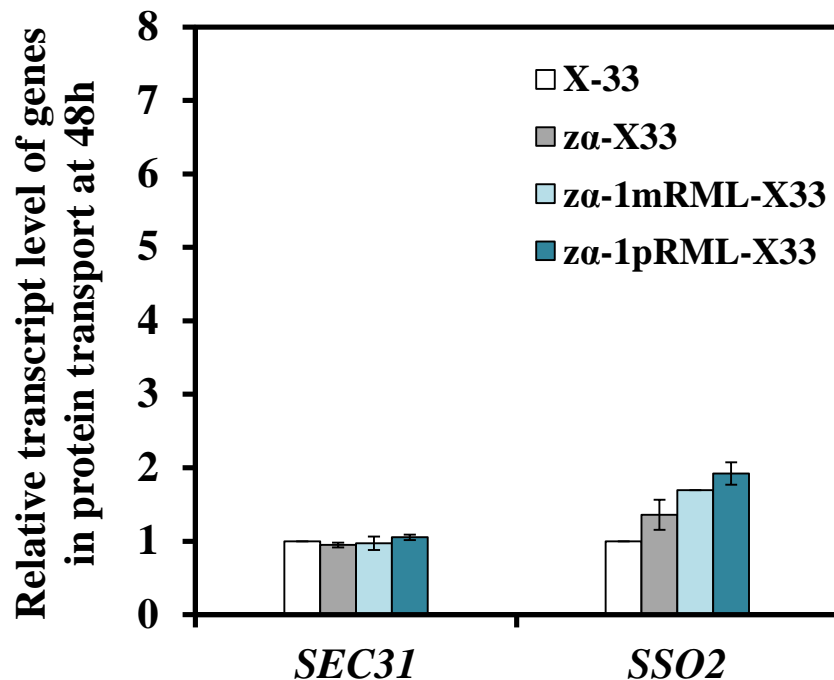**B**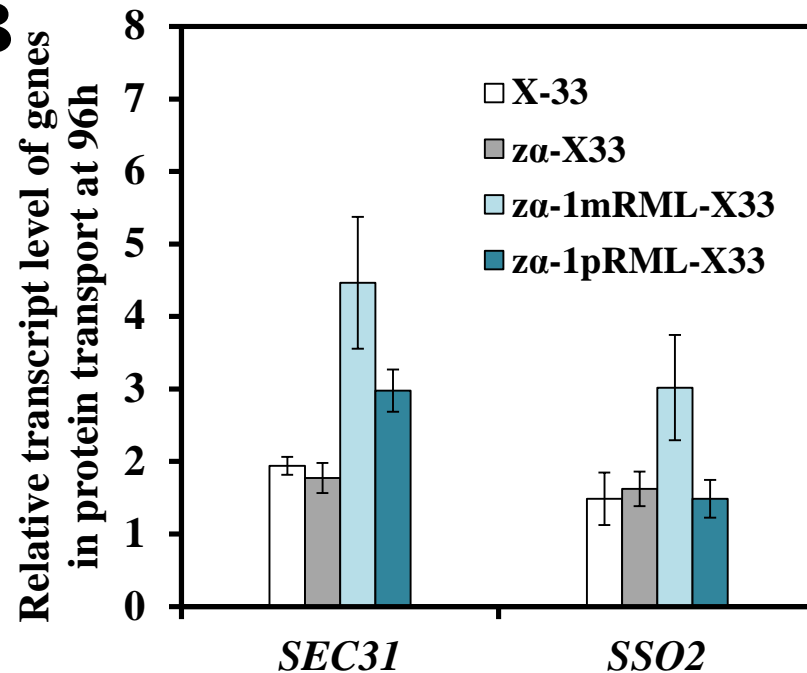**C**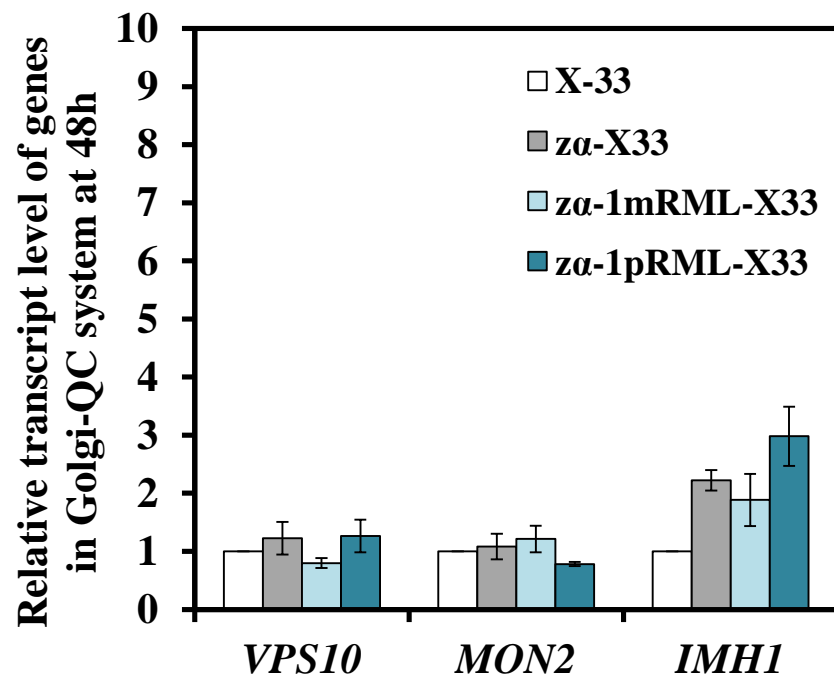**D**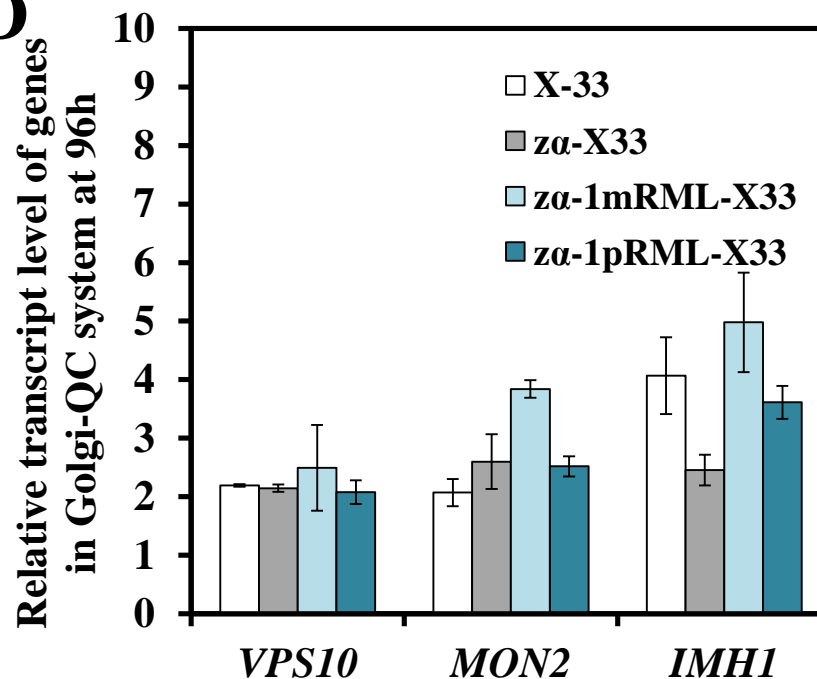

Supplement: Supplemental Material [file kbie-10-01-1614422-s003.pdf]
